# Search for Novel Diagnostic Biomarkers of Prostate Inflammation-Related Disorders: Role of Transglutaminase Isoforms as Potential Candidates

**DOI:** 10.1155/2019/7894017

**Published:** 2019-07-09

**Authors:** Maria Pia Savoca, Antonino Inferrera, Elisabetta A. M. Verderio, Daniela Caccamo

**Affiliations:** ^1^School of Science and Technology, Centre for Health, Ageing and Understanding of Disease, Nottingham Trent University, Nottingham NG11 8NS, UK; ^2^Department of Human and Paediatric Pathology “Gaetano Barresi”, Polyclinic Hospital University of Messina, Messina 98125, Italy; ^3^BiGeA, University of Bologna, Bologna 40126, Italy; ^4^Department of Biomedical Sciences, Dental Sciences and Morpho-Functional Imaging, Polyclinic Hospital University of Messina, Messina 98125, Italy

## Abstract

Investigations on prostate inflammation-related disorders, including acute and chronic prostatitis, chronic pelvic pain syndrome, benign prostate hyperplasia (BPH), and prostate cancer (PCa), are still ongoing to find new, accurate, and noninvasive biomarkers for a differential diagnosis of those pathological conditions sharing some common macroscopic features. Moreover, an ideal biomarker should be useful for risk assessment of prostate inflammation progression to more severe disorders, like BPH or PCa, as well as for monitoring of treatment response and prognosis establishment in carcinoma cases. Recent literature evidence highlighted that changes in the expression of transglutaminases, enzymes that catalyze transamidation reactions leading to posttranslational modifications of soluble proteins, occur in prostate inflammation-related disorders. This review focuses on the role specifically played by transglutaminases 4 (TG4) and 2 (TG2) and suggests that both isoenzymes hold a potential to be included in the list of candidates as novel diagnostic biomarkers for the above-cited prostate pathological conditions.

## 1. Introduction

Prostate is classified as an immune-competent organ, being populated by leukocytes, including stromal and intraepithelial T and B lymphocytes, macrophages, and mast cells; notably, the number of leukocytes increases with aging [1]. In retrospect, it is significant to note that reviews of immunobiological studies of the prostate and patients with diseases thereof (the latest of which is [2]), including observations and suggestion of the existence of a local immunological system, i.e., “prostate lymphoreticular system,” *ergo* “being classified as an immune-competent organ” [1], were made prior to more contemporary observations. In the last decades, clinical trials and epidemiological observations have shown that prostate inflammation is a common condition in aged men [3–5].

Prostate inflammation is classified through histological evaluation of the amount of inflammatory cells and their effect on prostate tissue [6]. The prostate inflammatory process may be triggered through different molecular pathways of the immune system, which are dysregulated secondarily to different factors: infectious bacterial agents, viruses, or other sexually transmitted microorganisms, urinary reflux, aging, dietary factors, hormones, autoimmune response, or a combination of these factors [1, 2, 7, 8].

Inflammation is typically distinguished as acute (neutrophilic cell infiltrate) and chronic (mononuclear cell infiltrate) [9]. Prostate inflammation may become a chronic process when the microorganism causing acute prostatitis is not eradicated.

Chronic prostatitis (CP) is a clinical syndrome, affecting 10% of the male elderly population, characterized by lower genitourinary tract and/or genital pain or discomfort and by inflammatory cells in prostatic secretions [10]. It has also been defined as CP/chronic pelvic pain syndrome (CP/CPPS) since it is not always caused by an infection. The presence of mononuclear inflammatory cells in the prostate is not diagnostic for CP/CPPS, and other inflammatory cells, such as T-cells, are commonly detected [4].

Chronic inflammatory processes are accompanied by the release of large amounts of proinflammatory cytokines, as well as oxygen and nitrogen reactive species (ROS and RNS). Oxidative stress plays a key role in the pathogenesis of CP/CPPS [11]. Cell response to both inflammation and oxidative stress involves the activation of transcription factor nuclear factor-kappa B (NF-*κ*B), leading to the upregulation of genes encoding for proinflammatory cytokines, the levels of which are further increased, as well as chemotactic factors and growth factors [12]. Several evidences show the presence of oxidative stress markers in the secretions of the genitourinary tract in patients affected by prostate inflammation [11].

Notably, chronic prostate inflammation is a process that could lead to the development of more severe prostatic disorders, like benign prostatic hyperplasia and prostate cancer, and also of the genitourinary tract [1, 2, 13–17].

Benign prostatic hyperplasia (BPH), the most common urological disorder in 70-80-year-old men [18], is a chronic progressive condition due to a nonmalignant propagation of stromal and epithelial cells eventually leading to prostate enlargement, associated with the development of nodules and proliferative inflammatory atrophy [19]. The pathogenesis and progression mechanisms of BPH are still not well characterized, but they likely involve several factors, i.e., changes in epithelial-stromal interactions, alterations of local endocrine and autonomous nerve system, tissue damage, and subsequent chronic tissue healing. Some authors have assimilated BPH to an immune-mediated inflammatory disease [13, 14, 20–22], others suggested a relationship between prostatic inflammation and lower urinary tract symptoms (LUTS) [3, 4] and/or other promotional factors common to BPH and PCa [14].

Prostate cancer (PCa) is considered as a chronic disease and the most prevalent cancer in the male population [23]. Epidemiological studies suggest that PCa is hormone-dependent and is associated with inflammation, as suggested by the detection of PCa susceptibility genes involved, such as RNAseL, MSR1, TLR4, MIC1, PON1, BRCA2, CHEK2, and OGG, which have a role in prostate carcinogenesis, and also in the host response to infection, inflammation, and oxidative stress [24, 25]. However, there is not a clear evidence showing that a reduction of PCa incidence and progression may be achieved through inhibition of prostate inflammation [26].

In the last years, great efforts have been undertaken to identify novel biomarkers that, alone or combined with the calculation of risks for the progression of prostate inflammation to BPH or PCa, may reduce the need for unnecessary biopsies, improve the stratification of low-risk patients, and better predict the response to treatments. Moreover, ideal biomarkers should also be able to accurately discriminate between indolent and aggressive cancers and identify men at high risk for developing PCa that require immediate treatment [27].

Several lines of evidence suggest that a key role in inflammatory processes and in the inflammation-induced progression of several types of cancers is played by transglutaminase 2 (TG2) or tissue transglutaminase. TG2 is an ubiquitous member of the transglutaminase (TG) family of enzymes, that catalyze calcium-dependent transamidation reactions through the incorporation of amines to glutamine residues (polyamination) or cross-linking of lysine residues to glutamine residues, resulting in the formation of intra- and intermolecular, covalent *ε*-(*γ*-glutamyl)lysine isopeptide bonds [28]. The involvement of TG2, and also transglutaminase 4 (TG4), another member of TGs that is uniquely distributed in the prostate gland, has been reported in BPH and PCa [29–34].

This review will summarize available literature data on biomarkers of prostate disorders and discuss the role of TG isoforms as potentially novel diagnostic biomarkers of prostate inflammation-related diseases.

## 2. Current Biomarkers of Prostate Disorders

The first biomarker of prostatitis condition was the white blood cell (WBC) count in prostatic secretion, but then, it was discarded due to its poor sensitivity [35]. The assessment of serum levels of prostate specific antigen (PSA), a member of the kallikrein gene family, is still carried out for PCa screening, even though the classification of PSA as a PCa biomarker has become increasingly controversial due to the lack of specificity [36]. Suspicious digital rectal examination and/or a serum PSA level ≥ 4.0 ng/mL, usually considered as criteria to prompt further evaluation for PCa [37], has been the subject of increasingly false positives and overdiagnosis. For instance, the increase of serum PSA levels has also been correlated with the aggressiveness of histological inflammation [38]. The low specificity of this test may be due to the presence of different PSA isoforms, since alternative splicing and alternative polyadenylation lead to the formation of at least 15 PSA transcripts, encoding for at least eight different proteins [39]. An investigation about the changes of these spliced variants in PCa is required for more specific PSA screening tests. Notably, several alternatively spliced isoforms of other kallikreins (KLK2, KLK3, KLK11, and KLK15) have been shown to be upregulated in PCa [40].

Chronic inflammation has been associated with the severity of lower urinary tract symptoms (LUTS), higher International Prostate Symptom Score (IPSS), and higher prostate volume if compared to prostate in normal conditions [41, 42]. Even though less invasive methods are used to assess the risk of developing prostatic chronic inflammation, such as detection of circulating biomarkers or imaging, the unique instrument to make a diagnosis of CP remains the histological exam [6, 43]. In prostate biopsies, the Chronic Prostatitis Symptom Index (CPSI) is utilised to highlight prostatitis-like symptoms, such as pain and burning sensation, dribbling and hesitant urination, urgency, or painful ejaculations. In fact, the total CPSI score is significantly associated with chronic inflammation. Moreover, patients with CP/CPPS are evaluated with UPOINT (urinary, psychosocial, organ-specific, infection, neurogenic, and tenderness) categorization [44].

A further diagnostic feature is the presence of prostatic calcifications, which increase progressively with aging, and the incidence of which has significantly been associated with greater inflammation and protracted symptoms in patients with CPPS [45–47].

One of the most reliable prostatic inflammation markers is the level of IL-8 in seminal plasma [48, 49], even in comparison with other cytokines [35, 50]. Another experimental marker of prostatic inflammation is the urinary level of inducible T-cell costimulator (ICOS) protein involved in cell signaling, immune response, and cell proliferation, that has been significantly associated with maximum uroflowmetry and postvoid residual urine volume [41]. Furthermore, the involvement of monocyte chemotactic protein-1 (MCP-1) has been hypothesized in the pathogenesis of CP and BPH, as its increase in prostatic secretions is associated with prostate volume enlargement and the expression of the macrophage marker CD-68 [51, 52].

CP has also been associated with autoimmune responses against prostate antigens, including prostatic acid phosphatase (PAP), prostate steroid-binding protein (PSBP), PSA, and other antigens in prostate homogenates and seminal plasma [53–55].

PCa may present with both aggressive and indolent forms. Given that low-grade prostate cancer is clinically insignificant and usually not associated with metastatic dissemination, a common practice for the management of patients with “low risk” or “favorable risk” is active surveillance. This practice is appropriate for men having PSA levels lower than 10 ng/mL and involves the periodic monitoring of patients through serial PSA testing, digital rectal exam, and biopsy sampling [56].

The hardest challenges in the clinical practice are the right selection of patients eligible for biopsy intervention, the differentiation of indolent tumors from those with an unfavorable prognosis, and the early recognition of higher-risk disease in patients initially diagnosed with low-risk disease and monitored by active surveillance. Current diagnostic biomarkers of PCa include blood-based tests, such as ProstateHealth Index® and 4K score®, other than PSA; urine sample-based tests, i.e., prostate cancer antigen 3 (PCA3), SelectMDx®, and ExoDx Prostate IntelliScore®; and tissue-based tests (ConfirmMDx®, Oncotype®, Prolaris®, and Decipher®) following biopsy, transurethral resection, or radical prostatectomy [27].

## 3. Role of Transglutaminase 4 in Prostate Disorders

Transglutaminase 4 (TG4) was discovered in the early 1990s from a human prostate cDNA library [57]. Its physiological role has firstly been defined in rodents as a regulator of copulatory plug formation [58], which is relevant for the fertilization, as it allows the seminal fluid to be kept in the vagina [59]. Via the incorporation of seminal proteins, such as uteroglobin, semenogelins, or polyamines, into sperm cell surfaces, TG4 suppresses sperm antigenicity in the female genital tract [60–62]. The typical TG substrates, polyamines, in particular putrescine, spermidine, and spermine, are secreted by the prostate gland and released in the seminal fluid. In mice and humans, seminal plasma is rich in spermidine and spermine of prostatic origin which may regulate at the urethra level the seminal clot formation during the ejaculatory process [58]. Seminal vesicles, thus even in secretions, have demonstrated intermediate TG activity and low polyamine amount. Notably, the level of protein-bound polyamines in ventral and anterior prostate suggests a higher extracellular activity of prostatic TG and polyamines. Furthermore, Km values of rat prostate TG for all the polyamines confirm the regulatory role of polyamines in the formation of the seminal clot [63]. Interestingly, the presence of autoantibodies against TG4 in association with human male infertility has recently been demonstrated, suggesting that TG4 plays a key role in the fertilization competence of spermatozoa [64, 65].

The role of TG4 in humans is still not well characterized. Human TG4 is a secretory protein having an expression pattern restricted to the prostate, where it has been found in luminal epithelial cells. TG4 is released in the seminal fluid and responds to androgen stimulation [66, 67]. In this regard, evidence has been provided that the retinoic acid receptor gamma (RAR-G) plays a major role in the regulation of TGM4 and that the presence of the androgen receptor (AR), but not its transcriptional transactivation activity, is critical for TGM4 transcription [68].

It has been reported that some human prostate cancer cell lines lack TG4 expression [66], even if a basal transcriptional activity of the promoter was observed [69]. Results from an analysis of TG4 transcripts in metastatic prostate cancer specimens indicate that TG4 expression was reduced in most of them [70]. The downregulation of TG4 expression in prostate cancer was confirmed by histological analyses of its distribution within sections from tumor-containing prostatectomy specimens and needle biopsies. The staining was restricted to luminal epithelial cells and not detected in basal epithelial or stromal cells. Moreover, TG4 staining was detected in high-grade prostatic intraepithelial neoplasia specimens but not in prostate carcinoma cells [67].

Other data have shown that TG4 knockdown is correlated with a lowered invasive ability of prostatic cancer cells [71]. TG4 expression showed a relatively wide profile in different prostate cancer cell lines and was strongly induced in the low invasive CA-HPV-10 prostate cancer cell line [71]. Thus, TG4 can be involved with the invasiveness of prostate cancer cells. A similar result was reported by Jiang and coworkers showing TGM4 overexpression in a human prostate tumor compared to normal tissue and a higher expression in high-Gleason score tumors [31].

In a mouse prostate cancer model generated by deletion of the prostate epithelium-specific tumor suppressor PTEN, TGM4 was rapidly downregulated and the dedifferentiation of the prostatic epithelium was quicker than in organ-confined human prostate cancers [72]. Other studies have revealed that TG4 overexpression in prostatic cells increases the adhesion of tumor cells to endothelial cells and decreases the barrier function of the latter. Moreover, TG4 sensitizes prostate cells to hepatocyte growth factor (HGF) [73].

**Figure 1:**
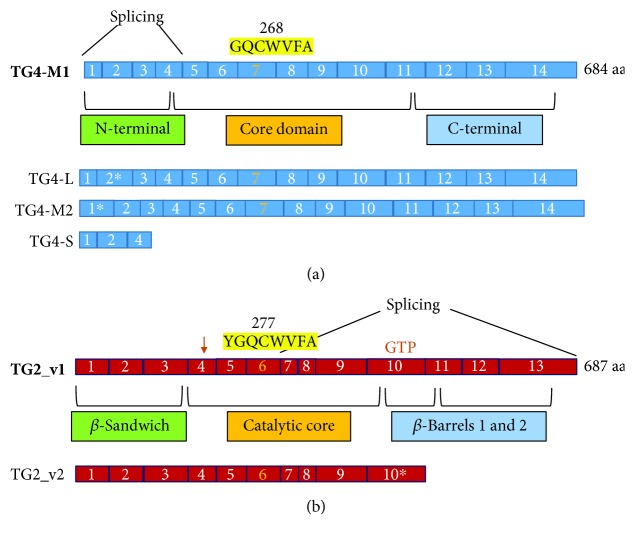
TG4 and TG2 alternative splicing. (a) Alternative splicing in TG4 transcript occurs at the N-terminal domain, leading to the formation of three variants. The asterisks indicate the exon interested by the splicing mechanism for each variant. (b) The sequence corresponding to the catalytic core or to the *β*-barrel domains of TG2 is involved in the splicing events. The intron retention mechanism that leads to the formation of TG2_v2 affects the exon 10. Shown here is the GTP/GDP binding site that involves also few amino acids (aa) in the exon 4 (arrow). In yellow is the conserved sequence of the catalytic core, including the cysteine (in bold).

These contradictory results may be explained taking into account that different studies may have addressed their efforts in the search for the classical TG4 form. However, proteins obtained by an alternative splicing mechanism acquire, in part, different structural and functional features compared to the original proteins [74]. These modifications are likely responsible for the failure of RT-PCR, Western blot, and immunohistochemical techniques aimed at detecting the classical TG4 form. Indeed, Cho and coworkers found four mRNA variants (L, M1, M2, and S) in the prostatic tissue of patients affected by BPH and PCa [32]. TG4-M1 and TG4-M2 have different splicing sites but not nucleotide size. Moreover, TG4-L, TG4-M1, and TG4-M2 have correct open reading frames, whereas TG4-S has a truncated reading frame (Figure 1(a)); however, the role of alternative splicing variants in prostate tissue is not well known.

Notably, TG4-M and TG4-S were detected in all tested BPH and PCa prostate tissues, while TG4-L was found in 56% of BPH and only in 15% of PCa. These findings suggest that changes in alternative splicing correlate with the development of PCa. However, TG4-L expression did not correlate with PSA serum levels, prostate volumes, or PSA densities [32]. Interestingly, although TG4 expressed in HeLa cells has been found as a secreted protein, TG4-L has not been detected in the supernatant of cell cultures [32]. The different cellular localizations suggest a different role for the spliced variants since they likely use different protein substrates, raising the idea that TG4 splicing variants may be exploited as diagnostic and/or therapeutic targets, as seen before for other genes in several cancers [75].

TGM4 expression levels were lower in moderately or poorly differentiated carcinoma compared with normal tissue [76]. Western blot analysis together with immunohistochemical analysis demonstrated TGM4 downregulation in prostate cancer [77]. Then, the comparison of protein markers in prostatic secretions of men having organ-confined tumors or extracapsular tumors showed differential expression of TG4, PSA, ANXA3, and matrix metalloproteinases (MMP7 and 9) between the two groups [78].

TG4 resulted also to be involved in cancer disease progression via promotion of epithelial-mesenchymal transition (EMT). In fact, in prostate epithelia-derived cancer cells, loss of E-cadherin, acquisition of N-cadherin, and cell migration have been highlighted [79].

## 4. Role of Transglutaminase 2 in Prostate Disorders

TG2 is a multifunction protein, since other than acting as a transamidating enzyme, it also displays GTPase, protein disulfide isomerase, kinase, cell adhesion, and scaffolding activities [80].

The function of TG2 in prostate is not fully understood yet. Unlike the prostate-specific TG4, TG2 is ubiquitous in prostatic tissue and is predominantly present in the intracellular compartment [57, 81]. Likely, TG2 is a pivotal component of cell signaling because it modulates the activation of the alpha1-adrenergic receptor, which mediates prostatic smooth muscle contraction [82].

**Figure 2:**
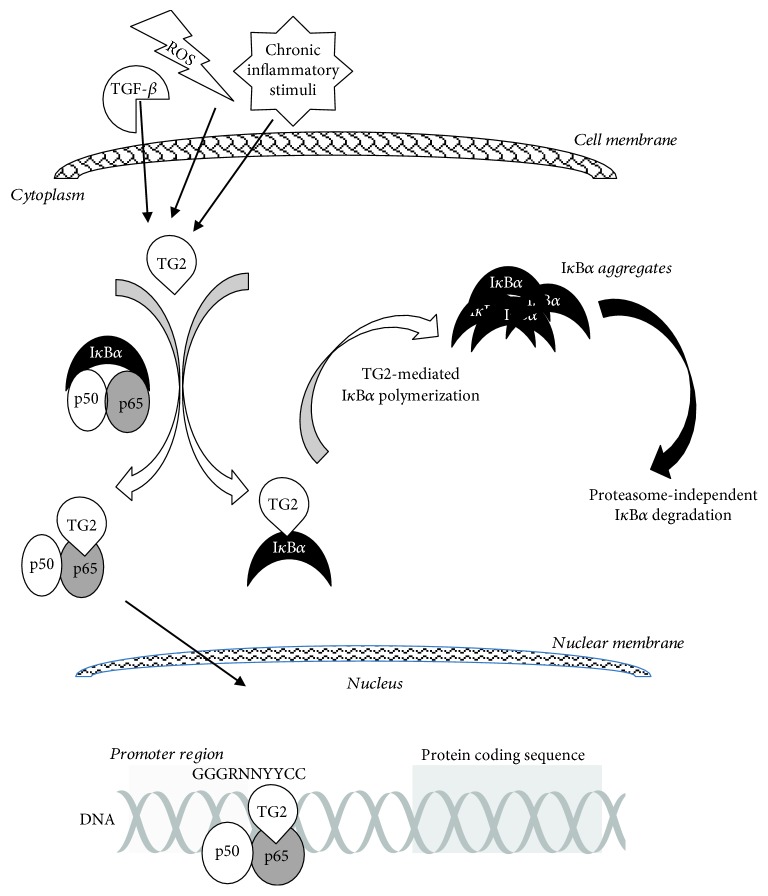
Schematic of the alternative NF-*κ*B activation pathway mediated by TG2. Inflammatory signals induce TG2 activation and interaction with the cytosolic inactive NF-*κ*B complex, composed of the transcriptionally active p50 and p65 subunits and the inhibitory I*κ*B*α* subunit. TG2 binding to I*κ*B*α* leads to I*κ*B*α* polymerization and release from NF-*κ*B complex, followed by degradation of I*κ*B*α* aggregates via a proteasome-independent pathway. The active p50/p65 heterodimer complex, associated with TG2, translocates to the nucleus where it binds to the NF-*κ*B consensus sequence (GGGRNNYYCC) present in the promoter region of several genes involved in inflammation as well as in tumorigenesis.

Numerous inflammatory cytokines and growth factors stimulate TG2 expression, such as IL-1, IL-6, TNF-*α*, and TGF-*β* [83–85], due to the presence in TGM2 gene promoter of different regulatory sites for inflammatory modulators, NF-*κ*B response element (-1338 bp), IL-6 specific *cis*-regulatory element (-1190 bp), and TGF-*β*1 response element (-868 bp). TG2 overexpression, in turn, has been reported to constitutively activate and extend NF-*κ*B activation through I*κ*B*α* polymerization and degradation (Figure 2), in chronic inflammatory conditions and in cancer cells [86–88].

TG2 protein and mRNA expression have been evaluated in experimental models of castration-induced prostatic atrophy with subsequent testosterone-induced prostatic hyperplasia. TG2 protein was strongly expressed and correlated with apoptosis. TG2 mRNA levels were not affected during the process of prostate involution but increased early in association with testosterone-induced proliferation [89]. Interestingly, in men treated with finasteride, a 5 alpha-reductase inhibitor, to reduce prostate size and ameliorate BPH symptoms, prostate involution has been shown to occur through both atrophy and cell death processes, in which TG2 has been demonstrated to play a relevant role [90].

Whey-acidic-protein four-disulfide core (WFDC) proteins play a major role as regulators of innate immunity, antimicrobial function, and inhibition of inflammatory proteases at mucosal surfaces. The prostate stromal 20 (ps20), a protein of the WFDC family, is a potent growth inhibition factor, which is also able to modulate the wound healing process and immune response. It has recently been shown that the posttranslational processing and cleavage of ps20 are required to generate a functional protein species and that TG2 mediates the cross-linking and cathepsin L cleavage to form components of a ps20 regulatory apparatus [91].

Cancer cells have been reported to express elevated levels of TG2; moreover, TG2 expression is further highly enriched in cancer-derived stem cells and promotes their survival [92]. However, conflicting results have been reported about the role of TG2 in PCa.

Despite the fact that TG2 was first suggested as a marker of apoptosis during treatment and progression of PCa [29], likely other mechanisms play a more relevant role in the prostate. Birckbichler and coworkers suggested that if all epithelial cells in prostate benign samples are immunoreactive for TG2, it is possible that the TG2 main function is not the promotion of apoptosis [93]. Interestingly, immunohistochemistry analysis demonstrated that TG2 expression is decreased in malignant glands as compared with benign or hyperplastic glands [93]. This study suggested the potential use of TG2 loss in prostate tissue as a potential biomarker for differentiation of PCa from BPH or other inflammatory conditions. Other than for diagnosis, the determination of TG2 levels could be useful for individual risk assessment and patient monitoring [93].

On the other hand, a strong interaction between protein kinase A anchor protein 13 (AKAP13) and TG2 has been reported in prostate cancer. Since AKAP plays a major role in protein kinase A and Rho protein-mediated signaling, the TG2-AKAP13 interaction has been suggested to play a role in prostate cancer [30].

Advanced prostate cancer is often associated with reduced androgen receptor (AR) expression leading to castration-resistant cancer [94]. In the physiological response to androgens, the AR binds to androgen-response elements (AREs) to modulate gene transcription. The transcription factor Oct1 is a context-dependent negative coregulator of AR. Notably, siRNA knockdown of Oct1 increases the transcription of TGM2, an endogenous AR target gene. Therefore, Oct1 may have regulatory functions in prostate development and cancer progression [95]. Recent studies demonstrated high TG2 basal levels in advanced prostate tumor cells showing a reduced AR expression. Notably, TG2 was shown to negatively regulate the AR level. In particular, TG2-mediated NF-*κ*B activation is able to induce NF-*κ*B binding to DNA elements in the AR gene, which in turn reduces AR gene expression [34].

Recently, an interesting role of TGM2_v2 (according to rationalized nomenclature [96]) or TG2 short form has been highlighted in prostate cell lines or specimens. TGM2_v2 mRNA is obtained from an alternative splicing event with an intron retention mechanism of intron X, and it encodes for a 548 aa (63 kDa) protein. The loss of a portion of the C-terminal domain (Figure 1(b)) decreases affinity for GTP, resulting in the escape from the negative control mediated by GTP on TG activity when there is a transient Ca^2+^ increase [97]. Interestingly, it has been reported that canonical and alternative TG2 isoforms are all expressed in normal human prostate tissue, even if the relative amount of TGM2 shorter transcript is small compared with a full-length one (TGM2_v2 10% vs. 90% TGM2_v1). Alternative splicing of TGM2 occurs differently in cancer cell lines, and in prostate cancer cells the alternative splicing of TG2 is a more active process. Indeed, the average expression levels of TGM2_v2 were found to be higher than that of the long classic transcript (TGM2_v1) in a small screening of prostate cancer tissues compared with normal tissues [96].

On the other hand, the differentiation treatment with *γ*-tocopherol of prostate carcinoma PC3 cells induced a decrease in the progression into the S-phase, which was associated with TG2 upregulation and increased activity, significant decrease of DNA synthesis, and protein expression of cyclins D1 and E. TG2 upregulation and activation could be part of a larger pathway that promotes the attenuation of prostate cancer malignancy [98].

## 5. Conclusions

In the last years, evidence has been provided that human TG4 and TG2 are alternatively spliced in prostate tissues and that alternative splicing processes occur in association with loss of prostate tissue homeostasis and transition towards pathological states, i.e., inflammatory conditions and cancer. This issue needs to be carefully addressed and become object of further investigations aimed at better understanding whether differential alternative splicing may represent a signature of specific prostate disorders. In fact, results from these investigations would open new diagnostic perspectives, particularly if TG4 and TG2, and their alternative variants, could be detected in serum samples from patients.

It is well known that TG4 is released in seminal plasma. Although the presence of TG4 in serum has not been investigated, the detection of autoantibodies against TG4 in the serum of male patients affected by infertility [64] suggests that TG4 could also move from seminal plasma to serum. If this was confirmed, the detection of TG4 and its alternatively spliced forms in the serum of patients with prostate disorders could be helpful in discriminating between prostate benign inflammatory conditions and malignant conditions. However, if the TG4 presence was only restricted to seminal plasma, the assessment of different TG4 variants in seminal plasma could be helpful for diagnostic purposes and represent an alternative to biopsy intervention.

TG2 is known to be exported outside the cells, and this event has been reported in inflammatory as well as malignant conditions [80]. However, its presence in the seminal plasma and serum has not yet been investigated. Given that TG2 autoantibodies are abundantly present in the serum of patients affected by celiac disease [99], it is not unreasonable to hypothesize that also TG2, and likely its alternatively spliced variants, may be present either in serum or in seminal plasma. The combined detection of TG4 and TG2 alternative variants in seminal plasma and serum could be a powerful tool for differential diagnosis of prostate disorders.
